# Investigating inhibitors of 1-deoxy-d-xylulose 5-phosphate synthase in a mouse model of UTI

**DOI:** 10.1128/spectrum.03896-23

**Published:** 2024-02-20

**Authors:** Eric C. Chen, Rachel L. Shapiro, Arindom Pal, David Bartee, Kevin DeLong, Davell M. Carter, Erika Serrano-Diaz, Rana Rais, Laura M. Ensign, Caren L. Freel Meyers

**Affiliations:** 1 Department of Pharmacology and Molecular Sciences, Johns Hopkins University School of Medicine, Baltimore, Maryland, USA; 2 Center for Nanomedicine at the Wilmer Eye Institute, Johns Hopkins University School of Medicine, Baltimore, Maryland, USA; 3 Department of Chemical & Biomolecular Engineering, Johns Hopkins University, Baltimore, Maryland, USA; 4 Department of Neurology, Johns Hopkins University School of Medicine, Baltimore, Maryland, USA; 5 Johns Hopkins Drug Discovery, Johns Hopkins University School of Medicine, Baltimore, Maryland, USA; 6 Department of Ophthalmology, Wilmer Eye Institute, Johns Hopkins University School of Medicine, Baltimore, Maryland, USA; 7 Department of Gynecology and Obstetrics, Johns Hopkins University School of Medicine, Baltimore, Maryland, USA; 8 Department of Infectious Diseases, Johns Hopkins University School of Medicine, Baltimore, Maryland, USA; 9 Department of Oncology, Johns Hopkins University School of Medicine, Baltimore, Maryland, USA; 10 Department of Biomedical Engineering, Johns Hopkins University, Baltimore, Maryland, USA; University of California, San Diego, La Jolla, California, USA

**Keywords:** 1-deoxy-d-xylulose 5-phosphate synthase, DXPS, antibiotic, central metabolism, MEP, acetylphosphonate, UTI

## Abstract

The rising rate of antimicrobial resistance continues to threaten global public health. Further hastening antimicrobial resistance is the lack of new antibiotics against new targets. The bacterial enzyme, 1-deoxy-d-xylulose 5-phosphate synthase (DXPS), is thought to play important roles in central metabolism, including processes required for pathogen adaptation to fluctuating host environments. Thus, impairing DXPS function represents a possible new antibacterial strategy. We previously investigated a DXPS-dependent metabolic adaptation as a potential target in uropathogenic *Escherichia coli* (UPEC) associated with urinary tract infection (UTI), using the DXPS-selective inhibitor butyl acetylphosphonate (BAP). However, investigations of DXPS inhibitors *in vivo* have not been conducted. The goal of the present study is to advance DXPS inhibitors as *in vivo* probes and assess the potential of inhibiting DXPS as a strategy to prevent UTI *in vivo*. We show that BAP was well-tolerated at high doses in mice and displayed a favorable pharmacokinetic profile for studies in a mouse model of UTI. Further, an alkyl acetylphosphonate prodrug (homopropargyl acetylphosphonate, pro-hpAP) was significantly more potent against UPEC in urine culture and exhibited good exposure in the urinary tract after systemic dosing. Prophylactic treatment with either BAP or pro-hpAP led to a partial protective effect against UTI, with the prodrug displaying improved efficacy compared to BAP. Overall, our results highlight the potential for DXPS inhibitors as *in vivo* probes and establish preliminary evidence that inhibiting DXPS impairs UPEC colonization in a mouse model of UTI.

**IMPORTANCE:**

New antibiotics against new targets are needed to prevent an antimicrobial resistance crisis. Unfortunately, antibiotic discovery has slowed, and many newly FDA-approved antibiotics do not inhibit new targets. Alkyl acetylphosphonates (alkyl APs), which inhibit the enzyme 1-deoxy-d-xylulose 5-phosphate synthase (DXPS), represent a new possible class of compounds as there are no FDA-approved DXPS inhibitors. To our knowledge, this is the first study demonstrating the *in vivo* safety, pharmacokinetics, and efficacy of alkyl APs in a urinary tract infection mouse model.

## INTRODUCTION

**Fig 1:**
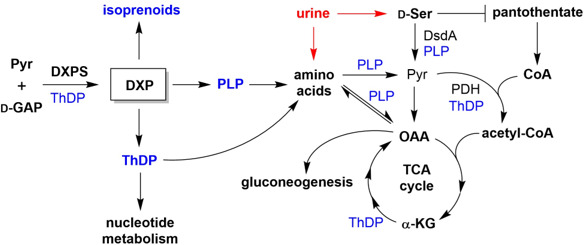
DXPS is a branchpoint enzyme that plays an essential role in bacterial adaptations important in the context of urinary tract infections. Urine provides peptides and amino acids as a main gluconeogenic carbon source to uropathogenic *Escherichia coli* (UPEC) (12–14). UPEC must metabolize toxins (e.g., d-Ser) to successfully adapt to the urinary tract.

New antibiotics are sorely needed to effectively combat rising rates of antimicrobial resistance. Progress has been made, with 19 new small-molecule antibacterial drugs approved between 2013 and 2022 (1). However, none of these are first-in-class drugs. It is crucial to identify new targets for the development of antibacterial agents with novel mechanisms of action (MoA) to slow the emergence of drug resistance. To this end, there is interest in exploring bacterial central metabolism as a target space. 1-Deoxy-d-xylulose 5-phosphate synthase (DXPS) is an enzyme in bacterial central metabolism that catalyzes the formation of DXP, a metabolite that is essential to bacteria and not found in humans. DXP sits at a metabolic branchpoint serving as a precursor in the biosynthesis of isoprenoids and the vitamins thiamin diphosphate (ThDP) and pyridoxal-5-phosphate (PLP) (2–5), all of which play critical roles in many aspects of bacterial growth and virulence (Fig. 1) (6–11). Thus, DXPS represents a promising antibacterial target in bacterial metabolism.

Bacterial pathogens readily remodel metabolism in order to adapt and colonize within fluctuating host environments (15–17). Different sites of infection demand different metabolic adaptations by pathogens. Thus, inhibiting bacterial processes required for the metabolic adaptation of a specific pathogen at a particular site of infection has the potential for the development of narrow spectrum treatment strategies. We have proposed that DXPS is critical for bacterial adaptation (6, 9), and urinary tract infection (UTI) provides an ideal system to investigate DXPS-dependent metabolic adaptations as potential targets for antibacterial development. Most UTIs are caused by uropathogenic *Escherichia coli* (UPEC) that originate from the gut (18). Compared to the gut, the environment of the urinary tract is nutritionally scarce, depleted of carbohydrates typically preferred by bacteria, and containing toxic metabolites and other host immune defenses that create a harsh environment for UPEC. UPEC have the capability to adapt to available nutrients and/or toxic metabolites (12, 13) by mechanisms thought to involve DXPS. For example, PLP is a critical cofactor enabling a diversity of reactions required by UPEC to utilize amino acids as a main carbon source in urine, and to detoxify the cell of the bacteriostatic metabolite d-serine (d-Ser), a host metabolite present in urine at high concentrations (13, 16, 19). Likewise, ThDP is crucial for a functioning TCA cycle and gluconeogenesis from amino acids in the urinary tract (12, 14). As both PLP and ThDP are derived from DXP, we hypothesize that DXPS is essential for these particular UPEC adaptations (Fig. 1).

Our previous work has produced DXPS-selective probes that enable studies of DXPS function for the development of therapeutic strategies targeting bacterial pathogen adaptation. Using butyl acetylphosphonate (BAP) (8, 20, 21), we showed that DXPS is required for the PLP-dependent breakdown of d-Ser in UPEC; BAP treatment sensitizes UPEC to the toxic effects of d-Ser, thus hindering this pathogen adaptation. UPEC grown in the presence of amino acids (including urine) were also shown to be particularly sensitive to inhibition of CoA biosynthesis in the presence of BAP, presumably due to the increased reliance on PLP-dependent processes and the TCA cycle for efficient gluconeogenesis (6). These preliminary studies suggested that treatment with a DXPS inhibitor has the potential to impede metabolic adaptation in the urinary tract and create specific metabolic vulnerabilities that can be co-targeted.

While these results are promising, experiments conducted in culture cannot fully recapitulate the fluctuating *in vivo* environment during infection. We have shown that changing the growth environment significantly alters susceptibility of *E. coli* to BAP in culture (6–8); however, the antimicrobial activity of alkyl acetylphosphonates (alkylAPs) against UPEC *in vivo* has not been assessed. In addition, toxicity, *in vivo* stability, and pharmacokinetic properties of DXPS inhibitors have not yet been evaluated. The goal of the present study is to advance alkylAPs as *in vivo* probes and assess the potential of inhibiting DXPS as a strategy to prevent infection *in vivo*.

Given our findings that DXPS plays an important role in UPEC adaptation, and BAP has activity against UPEC in urine, a relevant culture medium to UTI, we have selected UTI as an ideal *in vivo* infection model for these studies. First, there is a need to develop new strategies for the treatment of UTIs, which are among the most common infections, affecting >150 million people annually worldwide and primarily afflicting women (22, 23). Notably, UTI accounts for nearly 25% of all infections in women (24–26); the prevalence of UTI rises considerably with age, with 15%–20% of women aged 65–70 and 20%–50% of women over the age of 80 experiencing bacteriuria (27). This rise in prevalence is thought to be attributable, in part, to shifts in sex hormone production with the onset of menopause (18, 25). Second, the UTI mouse model has been considered a reasonable *in vivo* model to investigate antibiotic efficacy. The rodent bladder structure and cellular composition are reported to be reflective of the human bladder (28), and the mechanism of infection, invasion, and immune response are thought to recapitulate UTI in humans (28, 29). The increased prevalence of UTI in women, especially post-menopausal women, implicates a role of hormonal cycling. Consistent with this, mouse models of menopause and UTI correlate estrogen production with bacterial burden as ovariectomized mice exhibit greater urinary bacterial burden after inoculation (30).

Here, we take the first steps to investigate the inhibition of DXPS as a strategy to prevent infection in an *in vivo* prophylaxis model of UTI. We demonstrate a lack of overt toxicity of BAP at high doses in mice as well as favorable pharmacokinetic profiles of BAP and an alkylAP prodrug (31) for UTI. We also demonstrate a partial protective effect of BAP against UTI and improved efficacy with the more potent alkylAP prodrug. This proof-of-concept research is significant as it advances DXPS inhibitors as *in vivo* probes of this target and offers the first evidence that inhibition of DXPS can impair pathogen colonization *in vivo*.

## RESULTS

### An alkylAP prodrug is a potent inhibitor of UPEC growth in urine

**Fig 2:**
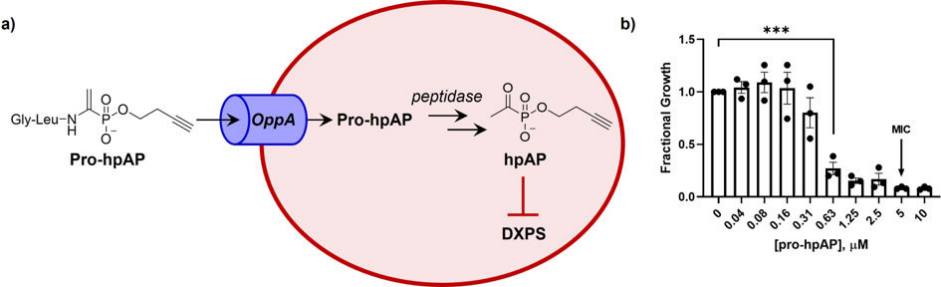
(a) Pro-hpAP is an alkylAP prodrug that enters bacteria via the OppA peptide transporter upregulated in UPEC during UTI. Once inside the cell, pro-hpAP undergoes intracellular activation via proteolysis to release the DXPS inhibitor hpAP; (b) Pro-hpAP inhibits CFT073 growth in urine with a MIC of 5 µM; error bars represent standard error, data for three biological replicates shown (•); *P* ≤ 0.001 (***).

The antimicrobial activity of BAP depends upon the culture conditions. We have shown that BAP is most potent under nutrient limitation, displaying MICs in the low micromolar range (7, 8). Higher potency in nutrient-limited compared to nutrient-rich conditions was found to be due, in part, to enhanced uptake into bacteria (7). BAP also inhibits the growth of the common UPEC strain, CFT073; however, the activity of BAP against CFT073 in urine, a nutrient-limited host environment, is lower compared to its activity in standard nutrient-limitation condition (6). In urine, a significant reduction in UPEC growth (~80%) is observed at 156 µM BAP, with higher BAP concentrations required to suppress growth >90% (MIC = 1250 µM). To address probe permeability in *E. coli*, we developed enamide prodrugs of alkylAPs that are designed to increase uptake through the peptide transporter OppA, known to be upregulated in UPEC during UTI (Fig. 2a) (32, 33). A prodrug of a structurally related alkylAP, homopropargyl acetylphosphonate (pro-hpAP), emerged as the most potent analog under standard nutrient limitation conditions (MIC ~20 nM) (31). Here, we have evaluated pro-hpAP against UPEC grown in urine (Fig. 2b). A significant reduction in fractional growth (>70%) was observed at 630 nM pro-hpAP, with higher concentrations required to suppress growth to >90% (MIC_pro-hpAP_ = 5 µM, Fig. 2b). Thus, pro-hpAP, exerting the same MoA as BAP but with significantly higher (250-fold) potency, is a potentially valuable probe to investigate DXPS in the context of UTI.

### BAP is well tolerated by mice

**Fig 3:**
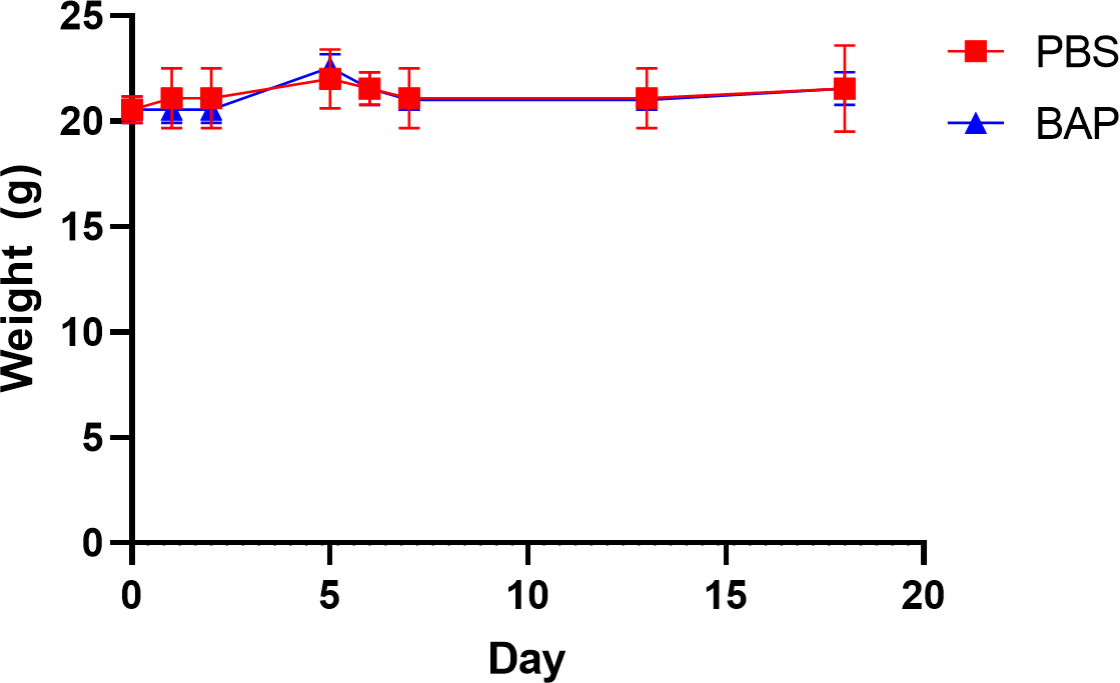
Acute safety studies show no signs of toxicity. Mice exhibit no symptoms of distress or change in weight with administration of the highest dose tested for BAP, 300 mg/kg (*n* = 2). Data are shown as mean ± SD.

To our knowledge, DXPS inhibitors have not been tested in mouse models, and their toxicity is unknown. While BAP has been shown to be selective for DXPS over other ThDP-dependent enzymes (20), the high concentration of BAP required to inhibit UPEC growth in urine culture increases the likelihood that high doses will be required for *in vivo* efficacy, which, in turn, have the potential to be toxic. Thus, as a first step, we conducted an *in vivo* dose-escalation study to assess for acute toxicity associated with alkylAP administration. Mice (*n* = 2) were administered a single dose of BAP at escalating doses up to 300 mg/kg (mpk), monitored for altered behavior, and weighed for 18 days. No overt toxicity or altered behavior was observed, and weight was maintained throughout the observation period for all doses administered (Fig. 3).

### Alkyl APs have favorable pharmacokinetic properties for UTI

Achieving *in vivo* efficacy with a chemical compound necessitates that it is stable enough to reach the site of infection and exert its effects, in this case, in the urinary tract. Thus, *in vitro* and *in vivo* pharmacokinetic (PK) studies were conducted to assess the stability of BAP and pro-hpAP in plasma and liver and evaluate their exposure in the urinary tract to inform on the viability of these compounds in mouse models of UTI.

**Fig 4:**
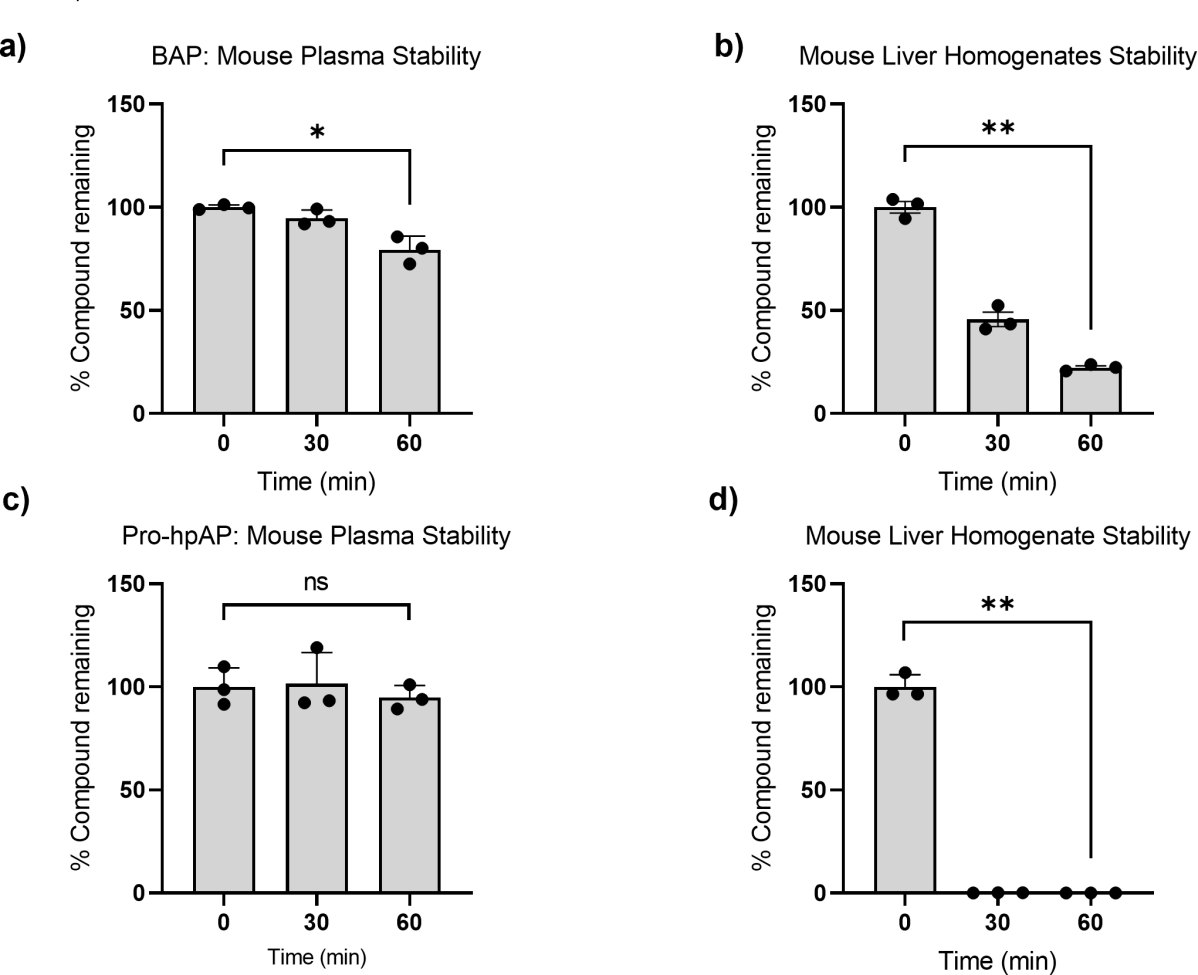
*In vitro* metabolic stability of BAP and pro-hpAP. (a) BAP is stable in plasma (b) but is metabolized in mouse liver homogenates. (c) Pro-hpAP is also stable in plasma (d) but is unstable in mouse liver homogenates. All experiments were performed in triplicate. Data are shown as mean ± SD, *P* ˃ 0.05 (ns), *P* ≤ 0.05 (*), *P* ≤ 0.01 (**).

BAP and pro-hpAP were incubated in mouse plasma, mouse liver homogenate (10%, wt/vol), and mouse liver microsomes (Fig. S1a). BAP demonstrated good stability in plasma (79% remaining at 1 h) and liver microsomes (94% remaining at 1 h); however, it was less stable in liver homogenate (22% remaining at 1 h), perhaps due to hydrolysis by esterase enzymes highly abundant in the liver (Fig. 4a and b; Fig. S1) (34, 35). Similarly, pro-hpAP also demonstrated good stability in plasma (95% remaining at 1 h) and liver microsomes (90% remaining at 1 h); however, it was highly unstable in liver homogenate with no prodrug remaining following 30 min incubation (Fig. 4c and d; Fig. S1).

**Fig 5:**
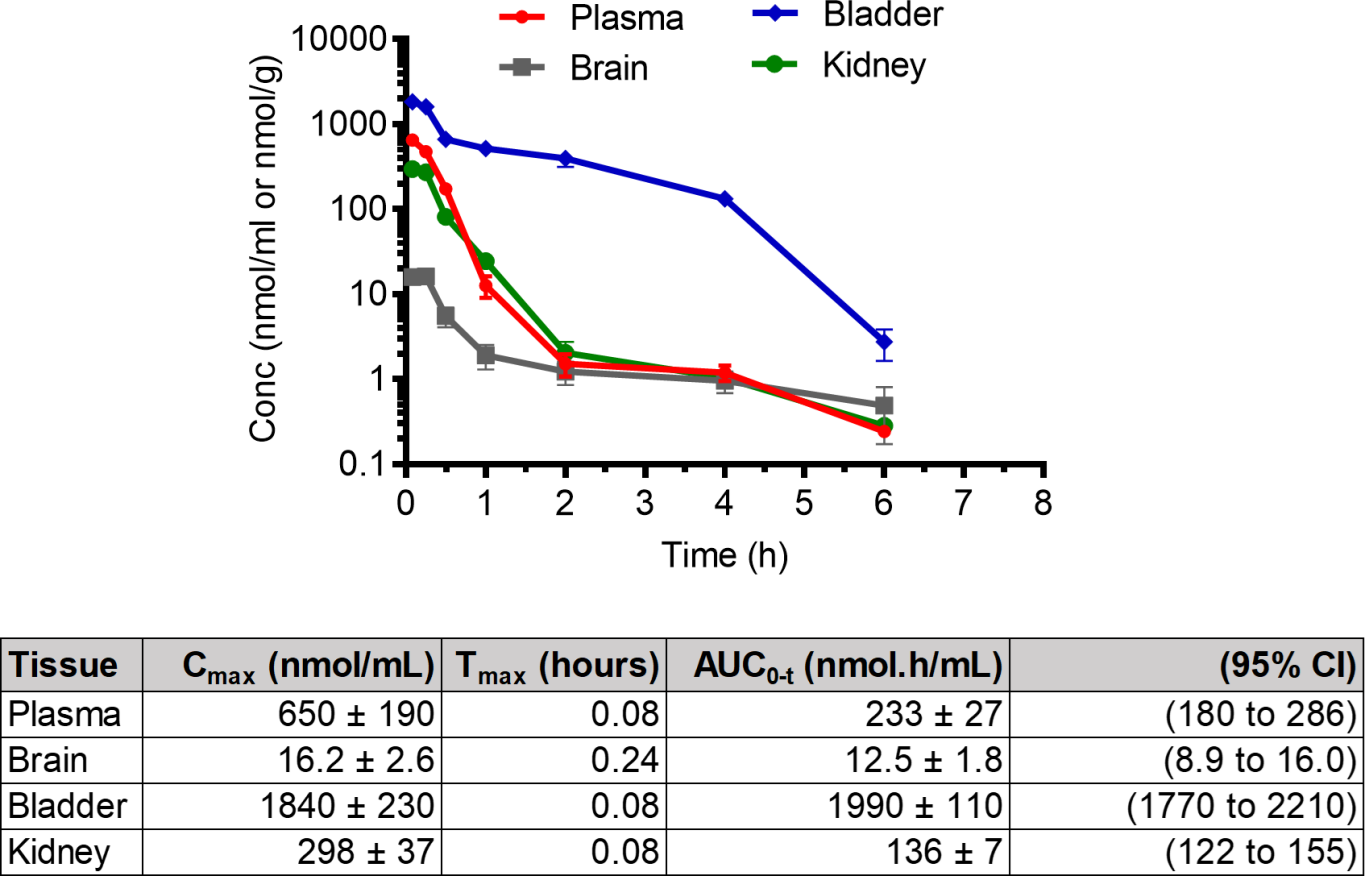
*In vivo* PK studies of BAP. PK profile for BAP was determined following a single 100 mg/kg IP dose; High concentrations were achieved in the bladder for all compounds. Four mice were used (two male, two female). Abbreviations: *C*_max_, maximal concentration after administration; *T*_max_, time to reach maximum concentration; AUC_0-t_, area under the concentration time curve from 0 to last measurable concentration; 95% CI, 95% CI for AUC_0-t_. Data are shown as mean ± SD.

**Fig 6:**
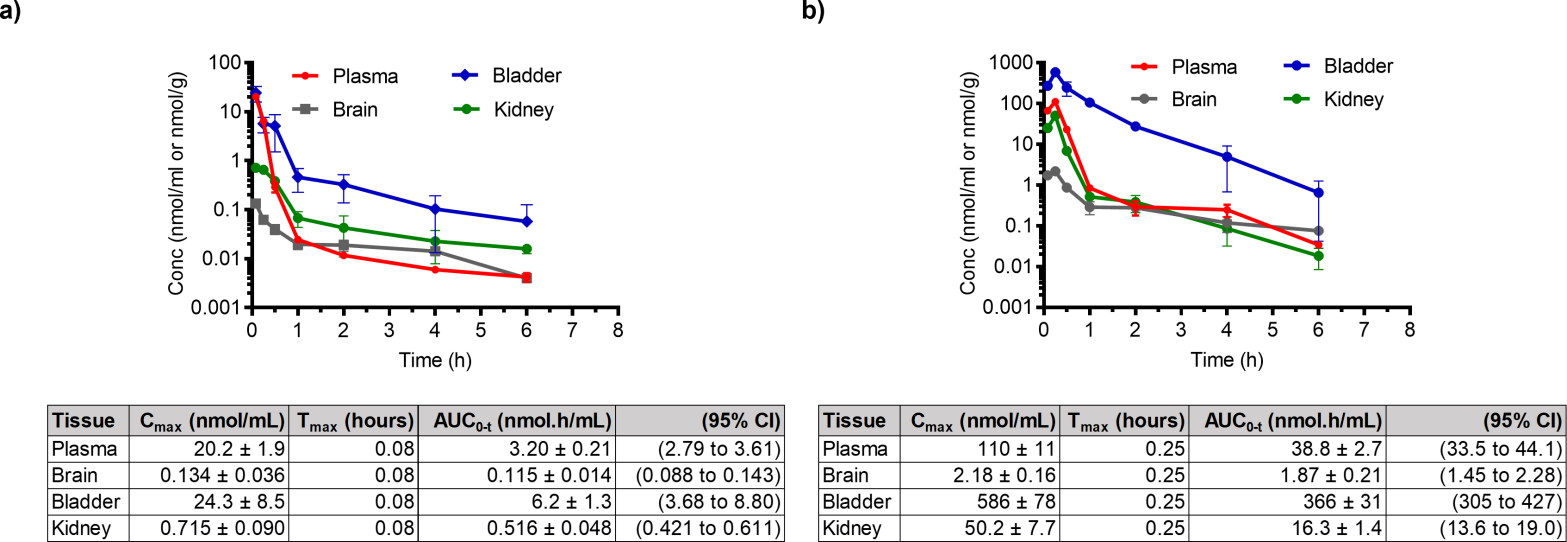
*In vivo* PK studies of pro-hpAP. (a) PK profile for pro-hpAP following a single 30 mg/kg IP dose; (b) hpAP release following a single 30 mg/kg dose of pro-hpAP. High concentrations were achieved in the bladder for all compounds. Four mice were used (two male, two female). Abbreviations: *C*_max_, maximal concentration after administration; *T*_max_, time to reach maximum concentration; AUC_0-t_, area under the concentration time curve from 0 to last measurable concentration; 95% CI, 95% CI for AUC_0-t_. Data are shown as mean ± SD.

*In vivo* pharmacokinetic analyses of BAP and pro-hpAP were conducted in CD-1 mice (*n* = 4, 2 male and 2 female per time point) (Fig. 5). Briefly, BAP was administered via IP injection (100 mg/kg) (Fig. 5), and BAP levels were quantified in plasma, bladder, kidney, and brain at predetermined time points using liquid chromatography with tandem mass spectrometry (LC/MS-MS) (Fig. 6). Pharmacokinetic profiles are illustrated in Fig. 5 and 6. BAP showed the highest exposures in the bladder (AUC = 1,990 ± 110 nmol h/g), followed by plasma and kidney (AUC = 233 ± 27 nmol h/mL and 136 ± 7 nmol h/g, respectively). Limited distribution was observed in the brain (AUC = 12.5 ± 1.8 nmol h/g), although low relative to other tissues (Fig. 5).

As peptide therapeutics are known to be excreted renally, through glomerular filtration (36), we reasoned that pro-hpAP could have high exposure in the urinary tract. While pro-hpAP was less metabolically stable than BAP in liver homogenate, we expected that peptide prodrugs may more readily distribute to the urinary tract and that it should be possible to achieve concentrations above MIC for this more potent analog. Pro-hpAP was administered by IP injection (30 mg/kg), and both pro-hpAP and released hpAP levels were quantified in plasma, bladder, kidney, and brain at 0.08–6 h post dose (Fig. 6a and b). Pro-hpAP exhibited the highest exposures in the bladder (AUC = 6.2 ± 1.3 nmol h/g, Fig. 6a), followed by plasma and kidney (AUC = 3.20 ± 0.21 nmol h/mL and 0.516 ± 0.048 nmol h/g, respectively). In this case, brain penetration was poor with negligible exposure observed (0.115 ± 0.014 nmol h/g). For hpAP released from pro-hpAP (Fig. 6b), the highest exposure was in the bladder (AUC = 366 ± 31 nmol h/g), followed by plasma and kidney (AUC = 38.8 ± 2.7 nmol h/mL and 16.3 ± 1.4 nmol h/g, respectively). Comparable to pro-hpAP, hpAP also displayed minimal exposure in the brain (AUC = 1.87 ± 0.21 nmol h/g) (Fig. 6b).

### BAP and pro-hpAP provide partial protection against infection in a mouse model of UTI

As a starting point to evaluate the efficacy of DXPS inhibitors *in vivo*, we conducted prophylaxis studies in a mouse model of UTI. In the ascending UTI model of infection, variability in infection burden has been noted (28, 37, 38). As the hormone cycle is implicated in UTI, and mice have a short estrous cycle lasting 4–5 days, we reasoned that exogenous synchronization may reduce the variability in bacterial burden. Mice naturally cycling in the estrus stage appeared to have greater variability in their infection burden compared to mice in the diestrus stage (Fig. S2). Thus, we opted to cycle arrest mice with a subcutaneous injection of medroxyprogesterone acetate (MPA) 1 week prior to the initiation of each prophylaxis experiment to induce a diestrus-like state.

**Fig 7:**
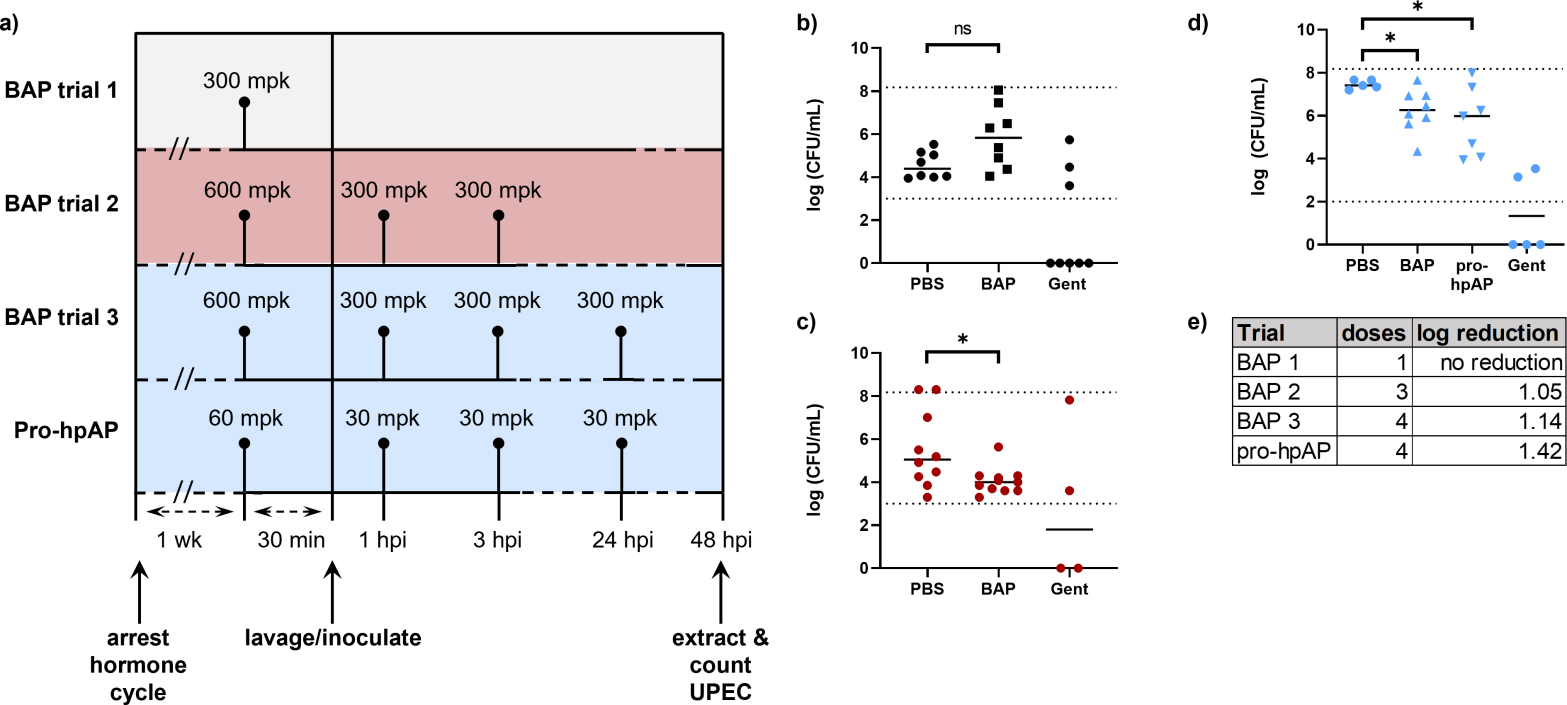
*In vivo* prophylaxis against UTI. (a) Dosing schemes for BAP (trials 1–3) and pro-hpAP. (b) A single high dose of BAP (300 mpk) prior to inoculation provides no protection against UTI; (c) increasing the initial BAP dose (600 mpk) with additional doses (300 mpk) at 1 and 3 hpi (red) partially protects against UTI; (d) an additional 300 mpk dose of BAP at 24 hpi has a modest effect to enhance protection against UTI. Pro-hpAP provides a similar level of protection against UTI as BAP at 10-fold lower doses (60 mg/kg prior to inoculation, 30 mg/kg 1, 3, and 24 hpi). (**E**) Table summarizing dosing and median log reduction of bacterial burden in AP-treated cohort compared to PBS-treated cohort. Each point depicts an individual mouse, with the line representing median log (CFU/mL); *P* > 0.05 (ns); *P* ≤ 0.05 (*). Dotted lines represent the limits of detection.

BAP has weak but measurable growth inhibitory activity against UPEC in urine and was observed to be well tolerated by mice (Fig. 3); thus, as a first step to evaluate BAP given prophylactically, we administered a single high dose of BAP (300 mg/kg) 30 min prior to UPEC inoculation. However, this single IP dose was not adequate to provide protection against UPEC infection, compared to treatment with the cidal agent gentamicin (10 mg/kg) which lowered bacterial burden to below the limit of detection (LOD) in 5 of 8 mice (Fig. 7b). Given the combined effects of BAP removal from the bladder through urination, and its short half-life, we reasoned that BAP may not be present at therapeutic levels for long enough in the urinary tract to exert protection against infection. Thus, we increased the dose to 600 mg/kg and administered additional IP doses at 1 and 3 h post infection (hpi). This presumably extended the time above MIC, which resulted in a 1.05 log reduction in bacterial burden, thus providing partial protection against UTI (Fig. 7c). Additional doses of BAP up to 30 hpi did not dramatically reduce bacterial burden further (1.32 log reduction compared to PBS control, Fig. S3).

The observation that BAP treatment achieved partial protection against UTI in mice is promising preliminary evidence suggesting that inhibiting DXPS impairs colonization. Since the enamide prodrug strategy effectively increases alkylAP potency in culture, we sought to determine if this strategy also enhances *in vivo* efficacy. Thus, we administered pro-hpAP to mice at a 10-fold lower dose compared to BAP, by IP injection 30 min prior to inoculation, with additional doses at 1, 3, and 24 hpi. Bacterial enumeration from mouse bladders showed that pro-hpAP reduced bacterial burden by 1.42 log at this lower dose, indicating pro-hpAP may be more potent in achieving partial protection against UTI (Fig. 7d).

## DISCUSSION

This work describes studies of the antibacterial target DXPS, an enzyme positioned at an essential metabolic branchpoint and poised to play critical roles in pathogen adaptation to nutrient-limited infection sites in the host. The urinary tract is one such host environment; UPEC must adapt to urine which is considered a nutrient-deplete, hostile condition (39, 40). Urine lacks preferred carbohydrate carbon sources and other nutrients, and contains toxic metabolites such as d-Ser. One response of UPEC is to shift metabolism to rely heavily on a functioning TCA cycle and gluconeogenesis to detoxify and/or utilize amino acids and peptides present in urine (12, 14, 41). Our previous work (6) has shown that UPEC require DXPS for adaptation to d-Ser, and blocking DXPS activity through BAP treatment creates a growth medium-dependent metabolic vulnerability that can be co-targeted. Hence, our studies to date highlight UTI as a suitable model to explore DXPS functions in pathogen adaptation and to develop new antibacterial approaches targeting DXPS-dependent adaptations.

In the present study, we have taken the first steps to evaluate *in vivo* PK properties of DXPS inhibitors and to investigate the effect of inhibiting DXPS on preventing infection *in vivo* by an ascending UTI assay. BAP was selected for these studies because of ease of access, its known selectivity for DXPS, and demonstrated antimicrobial effects against UPEC in urine (6, 20, 21). Here, our results from acute toxicity studies indicate BAP is well-tolerated by mice, suggesting we can safely target DXPS at high doses. In addition, *in vitro* and *in vivo* PK studies indicate that BAP reaches high concentrations in the bladder and kidneys despite rapid metabolism by non-CYP mediated enzymes. A more potent alkylAP prodrug, pro-hpAP, which is designed to increase cellular uptake via the OppA peptide transporter, inhibits UPEC growth in urine with significantly higher potency relative to BAP. Similar to BAP, pro-hpAP and released hpAP reach high concentrations in the bladder, suggesting good exposure in the urinary tract despite prodrug instability in mouse liver homogenates. These findings are consistent with previous reports that therapeutic peptides are renally excreted (36), including the phosphonopeptide antibiotic Alafosfalin, which is reported to accumulate in the urine through glomerular filtration (42–44).

We sought to prove the concept that therapeutic intervention at this metabolic branchpoint has the potential to prevent UTI, either by hindering metabolic adaptation or by other mechanisms that reduce the ability of UPEC to colonize the urinary tract. Thus, BAP and pro-hpAP were evaluated by the ascending UTI assay. Mice and humans have different hormone cycles, with mice having a short, 4–5 day estrous cycle during which there are sharp hormone fluctuations which could impact colonization (30, 45) and, thus, introduce variability in bacterial burdens in this assay. To attempt to limit variability arising from hormonal fluctuations, we arrested mice at diestrus stage with subcutaneous injections of MPA before transurethral inoculation with bacteria. In this model, BAP treatment 30 min prior to inoculation and up to 3 h post-inoculation reduces bacterial burden in mouse bladders by 1.05–1.32 log compared to the PBS control, with additional maintenance doses of BAP having only a minimal effect to reduce bacterial burden. Pro-hpAP treatment at a 10-fold lower dose relative to BAP had the effect to reduce bacterial burden comparably to BAP at high dose, consistent with the higher potency of pro-hpAP and suggesting this peptide-containing prodrug was delivered to and maintained in the murine urinary tract.

Factors related to bacterial titer and growth environment may contribute to the weak potency of BAP in this murine model. It is thought that in humans, pathogens originating from the gut colonize the periurethral surface as well as the vagina, from which they can ascend through the urethra into the urinary tract to cause UTI (46–50). In contrast, mice are not natural hosts for UPEC. Establishing infection in this model requires a high inoculum of UPEC (~10^8^ CFU) delivered directly into the bladder, significantly higher titers relative to UPEC present at the onset of UTI in humans (29, 51, 52). Thus, it is possible that the high titers required to establish infection give rise to an inoculum effect in the murine model, increasing the concentration required for DXPS inhibitors to prevent colonization in the bladder. In contrast, gentamicin is known to exert cidal post-antibiotic effects and, thus, may continue to lower bacterial burden after it falls to sub-therapeutic levels (53–57). It is also possible that the growth environment of the inoculum influences BAP potency. In this assay design, the inoculum is prepared in nutrient-rich LB broth, a condition in which bacterial uptake of alkylAP DXPS probes is limited, and BAP and pro-hpAP are significantly less potent (7, 8). While it is not known how microbial metabolism and sensitivity to BAP might shift when UPEC transition from the environment of periurethral and vaginal reservoirs to the urinary tract, LB is unlikely to recapitulate these environments in any case. Future research could seek to address this challenge for model studies toward developing DXPS as an antibacterial target. Overall, this work is significant because it provides the first insights from *in vivo* studies of DXPS-selective probes, demonstrating they are well tolerated by mice and exhibit favorable PK and offering promising preliminary evidence that inhibition of DXPS has an effect to impair colonization of a pathogen *in vivo*.

## MATERIALS AND METHODS

### Materials

Unless otherwise noted, reagents were obtained from commercial sources. BAP and pro-hpAP were synthesized according to previously published methods (20, 31). Gentamicin was obtained from Sigma-Aldrich Chemical Company (St. Louis, MO, USA). Pooled human urine was obtained from Innovative Research, Inc. (Novi, MI, USA) or Lee BioSolutions, Inc. (Maryland Heights, MO, USA). LB (BP1426-2) was purchased from Fisher Scientific (Waltham, MA, USA) and autoclaved. Agar (9002-18-0) was purchased from Millipore (St. Louis, MO, USA). Medroxyprogesterone acetate (MPA) injectable suspension was sourced from Auromedics. 30G needles (REF305106) and 1 mL syringes (REF309659) were purchased from BD. Catheter polyethylene tubing with 0.28 mm inner diameter (REF427401) was purchased from BD Intramedic. Optixcare eye lube used was purchased from Amazon. PBS (REF10010023) was purchased from Thermofisher (Waltham, MA, USA) and filter sterilized.

### General methods and instrumentation

Pooled human urine was filter-sterilized prior to use. Solid urine agar plates were prepared by supplementing cooled 1.5% (wt/vol) agar with filter-sterilized urine in a 4:1 ratio. OD_600_ measurements were performed on a Beckman DU800 UV/Visible spectrophotometer (Brea, CA, USA). MIC determination data were collected from bacteria in 96-well plates (Nunc Edge 96-Well, Non-Treated, Flat-Bottom Microplate) incubated and read on a Biotek Epoch 2 microplate reader. Uropathogenic *E. coli* strain CFT073 (ATCC: BAA-2503) was obtained from the lab of Rodney Welch (58). All microbial manipulation of pathogenic bacteria was conducted in a certified biosafety level 2 laboratory following all associated safety protocols. A hand-held tissue homogenizer (purchased from IKA, T10 basic S001, 3737001) was used to homogenize mouse bladders for bacterial enumeration.

### Animal studies

All animals were cared for and experiments conducted in accordance with protocols approved by the Animal Care and Use Committee of the Johns Hopkins University, and in compliance with the National Institutes of Health guidelines for the Care and Use of Laboratory Animals. Only female mice were inoculated transurethrally with bacteria as UTI predominantly effects females in the population and transurethral catheterization of male mice is difficult given the structural differences of the male urinary tract (59).

### Antimicrobial susceptibility

UPEC (CFT073) cells from frozen glycerol stocks were streaked onto urine agar plates and grown for 48 h at 37°C. Three to five isolated colonies were inoculated into filter-sterilized urine supplemented with FeSO_4_ (20 µM), and the cultures were grown overnight with shaking at 37°C. The saturated cultures were subcultured (1:50) in fresh urine supplemented with FeSO_4_ (20 µM) and grown to exponential phase (OD_600_ ~ 0.25). Cultures at exponential phase were diluted 1:1,000 into fresh urine supplemented with FeSO_4_ (20 µM) to yield the experimental inoculum which was mixed 1:1 with urine containing pro-hpAP at 2 × the desired final concentration (0–10 µM). The final concentration of bacteria in each well was ~10^5^ CFU/mL in a volume of 200 µL. Colony counts of the experimental inoculum were independently verified by dilution and enumeration on LB agar, grown for 16 h at 37°C, to confirm consistency between experiments. The 96-well plates were incubated at 37°C for 16 h with intermittent shaking. Fractional growth of cells treated with pro-hpAP was determined at 8 h relative to the no drug control. MIC determination was performed in biological triplicate.

### Acute toxicity studies

Six-week-old female FVB mice were administered BAP by intraperitoneal (IP) injection at doses of 1, 3, 10, 30, 100, and 300 mg/kg. The mice were observed every hour for 6 h post administration for signs of overt toxicity or mortality, or for symptoms related to pain/distress (ruffled fur, discharge from the eyes, dehydration, hunched posture, convulsions, lethargy, reluctance to move, uncoordinated movements, hypothermia, pale ears/feet, labored respiration, and/or cyanosis). Following initial observations, 6 h post administration, mice were then observed daily for signs of toxicity for 18 days. Mice that were administered the highest dose of BAP tested (300 mg/kg) were weighed at days 0, 1, 2, 5, 6, 7, 13, and 18.

### *In vitro* stability studies

*In vitro* stability studies were conducted using our previously published methods naive CD1 mouse plasma or liver homogenate. For liver homogenate stability study, washed mouse liver was diluted 10-fold in 0.1 M potassium phosphate buffer and homogenized using probe sonication. Crude homogenate was then aliquoted to 1 mL, and the homogenate was spiked with 10 µM compound. The plasma stability of the compounds was determined by spiking (10 µM) in 1 mL of plasma and incubating in an orbital shaker at 37°C. All the stability studies were conducted at predetermined times (0, 30, and 60 min), where 100 µL aliquots of the mixture in triplicate were removed and the reaction was quenched by the addition of three times the volume of ice-cold acetonitrile spiked with the internal standard (losartan: 0.5 µM). The samples were vortex-mixed for 30 s and centrifuged at 10,000*g* for 10 min at 4°C. An amount of 50 µL of the supernatant was diluted with 50 µL of water and transferred to a 250 µL polypropylene vial sealed with a Teflon cap. Compound disappearance was monitored over time using liquid chromatography and tandem mass spectrometry (LC–MS/MS). Paired *t*-tests were used to determine statistical significance between 0 and 60 min time points.

### *In vivo* PK studies

For pharmacokinetic analysis, both male (*n* = 2) and female (*n* = 2) CD1 mice were dosed with either BAP or pro-hpAP by intraperitoneal (IP) route or administration (100 mg/kg) and (30 mg/kg), respectively. Blood and tissue samples were collected at predetermined time points (0–6 h post-dose). Plasma was generated from blood by low-speed centrifugation (3,000 × *g*), and all samples were stored at −80°C until bioanalysis.

Non-compartmental-analysis module in WinNonlin was used to assess blood pharmacokinetic parameters including max concentrations (*C*_max_), time to *C*_max_ (*T*_max_), area under the curve (AUC), area extrapolated to infinity (AUC_0–∞_), and terminal half-life (*t*_1/2_). In addition, the tissue penetration index was measured as the ratio of AUC_tissue_ to AUC_plasma_.

To quantify BAP and pro-hpAP, methanol containing 0.5 µM losartan as an internal standard was added 5 µL/µL plasma or 5 µL/mg tissue in microcentrifuge tubes. Tissue was further homogenized using a Spex Geno/Grinder with stainless steel beads for 3 min at 1,500 RPM. Homogenates from untreated animals were spiked with the analytes from 100 to 0.01 nmol/g tissue by serial dilution to generate a standard curve. Plasma and tissue homogenates were then vortexed and centrifuged (16,000 × *g* for 5 min at 4°C) to precipitate proteins. Supernatants were transferred to a 96 well plate and 2 µL was injected on a Accela 1250 LC coupled to a Vantage triple quadrupole mass spectrometer (Thermo Fisher Scientific Inc., Waltham MA). Samples were separated on an Agilent EclipsePlus C18 RRHD (1.8 µm) 2.1 × 100 mm column with mobile phases consisting of water +0.1% formic acid (A), and acetonitrile + 0.1% formic acid (B). Separation was achieved at a flow rate of 0.4 mL/min using a gradient run. Samples were introduced to the source through heated ion spray with the capillary temperature setting at 350°C, spray voltage of 4 kV and S-Lens RF of 51. Nitrogen was used as the sheath and auxiliary gas with the settings of 50 and 1 arbitrary units, respectively. For BAP, the instrument was operated in negative ion mode scanning for transitions of 179.1 > 63.1, 79.1 (BAP) and 420.9 > 179.1, 127.0 (losartan) which were acquired with collision energies setting of 17; 55 and 25; 41 CE, respectively. For pro-hpAP, the instrument was operated in positive ion mode scanning for transitions of 346.2 to 86.1 and 143.2 (pro-hpAP) and 422.9 to 207.1 and 180.1 (losartan) which were acquired with collision energies setting of 29; 17 and 37; 22 CE, respectively. Peaks were quantified with Xcalibur software.

### Cycle arresting mice at diestrus stage

Female mice were injected subcutaneously in the flank with 2.5 mg of MPA (150 mg/mL, diluted in sterile saline) 7 days prior to inoculation (60). Mice were dosed vaginally with 30 µL of sterile saline lavage fluid immediately prior to inoculation. The fluid was collected, placed on a slide, heat fixed, Gram stained, and assessed to determine the estrous cycle stage of each mouse as previously described (61).

### Evaluating BAP and Pro-hpAP in the *In Vivo* ascending UTI assay

Six-week-old female C3H/HeN mice were inoculated with UPEC as previously described (28). Briefly, UPEC strain CFT073 (ATCC designation: BAA-2503) was streaked on LB agar from a frozen glycerol stock and grown overnight at 37*°*C. Following overnight incubation, three to five colonies were picked and subcultured into 10 mL of sterile LB, and the culture was incubated statically overnight at 37*°*C. The culture was diluted 1:1,000 (25 µL of culture into 25 mL of sterile LB) and incubated statically for 24 h at 37°C to promote the growth of bacteria with type-1 pili expression. The resulting 25 mL inoculum was centrifuged at 4,200 × *g* for 20 min at 4°C. The supernatant was removed, and the cell pellet was resuspended in 10 mL of sterile PBS. The OD_600_ was adjusted to 1.0 by the addition of PBS to give a cell density of approximately 4 × 10^7^ to 1 × 10^8^ CFUs per 50 µL, the volume of the inoculum. Cell counts were validated by plating a dilution of the inoculum solution and enumerating following overnight incubation.

Urinary catheters were assembled by threading 30G needles with 0.28 mm inner diameter polyethylene tubing to create a soft tip. Catheter tips were dipped in lubricant immediately before inoculation. During catheterization, urine was aspirated from the bladder prior to inoculation with 50 µL of UPEC inoculum, prepared as described above. Mice were housed with their drug-dosed cohort and dosed intraperitoneally according to each experiment’s dosing regimen as shown in the figures. Dosing regimen for BAP trial 1 (Fig. 7b)consisted of IP administration of BAP (300 mg/kg) 30 min before inoculation; dosing regimen for BAP trial 2 (Fig. 7c) consisted of IP administration of BAP (600 mg/kg) 30 min before inoculation followed with IP doses of BAP (300 mg/kg) at 1 and 3 hpi; dosing regimen for BAP trial 3 and the pro-hpAP trial (Fig. 7d) consisted of IP administration of BAP (600 mg/kg) or pro-hpAP (60 mg/kg) 30 min before inoculation followed with IP doses of BAP (300 mg/kg) or pro-hpAP (30 mg/kg) at 1, 3, and 24 hpi; dosing regimen 4 (Fig. S3) consisted of IP administration of BAP (600 mg/kg) 30 min before inoculation followed with IP doses of BAP (300 mg/kg) at 1, 3, 6, 10, 22, 26, and 30 hpi. Gentamicin diluted in sterile PBS was used as a positive control and was administered to mice at 10 mg/kg IP 30 min prior to inoculation in all experiments.

After 48 h, mice were anesthetized by isoflurane inhalation and sacrificed by cervical dislocation. Bladders were harvested, added to 1 mL sterile PBS, and homogenized with a hand-held homogenizer in a biosafety cabinet. Tissue homogenates were serially-diluted (five 10-fold dilutions), and 100 µL of each dilution was plated onto LB plates and enumerated after a 24 h incubation at 37°C, yielding an effective detection range of 1 × 10^2^–150 × 10^8^ CFU/mL. In a few cases, bacterial counts fell below the lower limit of detection or above the upper limit of detection and are reported as 0 CFU/mL or conservatively 200 × 10^8^ CFU/mL, respectively.

## SUPPLEMENTAL MATERIAL

Supplemental material spectrum.03896-23-s0001.docx Figures S1 to S3.

ASM does not own the copyrights to Supplemental Material that may be linked to, or accessed through, an article. The authors have granted ASM a non-exclusive, world-wide license to publish the Supplemental Material files. Please contact the corresponding author directly for reuse.
