# Supplementary material for: Investigating inhibitors of 1-deoxy-d-xylulose 5-phosphate synthase in a mouse model of UTI
Source: Microbiol Spectr. 2024 Feb 20;12(4):e03896-23. doi: 10.1128/spectrum.03896-23 (PMC10986598; doi:10.1128/spectrum.03896-23)
Supplement: Supplemental material — Figures S1 to S3. [file spectrum.03896-23-s0001.docx]

**Investigating inhibitors of 1-deoxy-d-xylulose 5-phosphate in a mouse model of UTI**

Eric C. Chen,^1^ Rachel L. Shapiro,^2^ Arindom Pal,^3,4^ David Bartee,^1^ Kevin DeLong,^2^ Davell M Carter,^1^ Erika Serrano-Diaz,^1^ Rana Rais,^1,3,4^ Laura M. Ensign,^5^ Caren L. Freel Meyers^1^

1. Department of Pharmacology and Molecular Sciences, The Johns Hopkins School of Medicine, Baltimore, MD, USA
2. Center for Nanomedicine at the Wilmer Eye Institute, Johns Hopkins University School of Medicine, Baltimore, MD, USA; Department of Chemical & Biomolecular Engineering, Johns Hopkins University, Baltimore, MD, USA.
3. Department of Neurology, The Johns Hopkins School of Medicine, Baltimore Maryland, USA
4. Johns Hopkins Drug Discovery, The Johns Hopkins School of Medicine, Baltimore Maryland, USA
5. Center for Nanomedicine at the Wilmer Eye Institute, Johns Hopkins University School of Medicine, Baltimore, MD, USA; Department of Ophthalmology, Wilmer Eye Institute, Johns Hopkins University School of Medicine, Baltimore, MD, USA; Department of Pharmacology and Molecular Sciences, Johns Hopkins University School of Medicine, Baltimore, MD, USA; Department of Chemical & Biomolecular Engineering, Johns Hopkins University, Baltimore, MD, USA; Departments of Gynecology and Obstetrics, Infectious Diseases, and Oncology, Johns Hopkins University School of Medicine, Baltimore, MD, USA; Department of Biomedical Engineering, Johns Hopkins University, Baltimore, MD, USA.

*Corresponding author: [cmeyers@jhmi.edu](mailto:cmeyers@jhmi.edu)

**Table of Contents**

page

**Figure S1:** Values of % compound remaining of in vitro PK…………………………………………………...……….2

**Figure S2:** Bacterial burden correlated to estrous cycle of mice……………………………………………..………..3

**Figure S3:** Higher frequency dosing of BAP effect on bacterial burden……………………………………..………..4


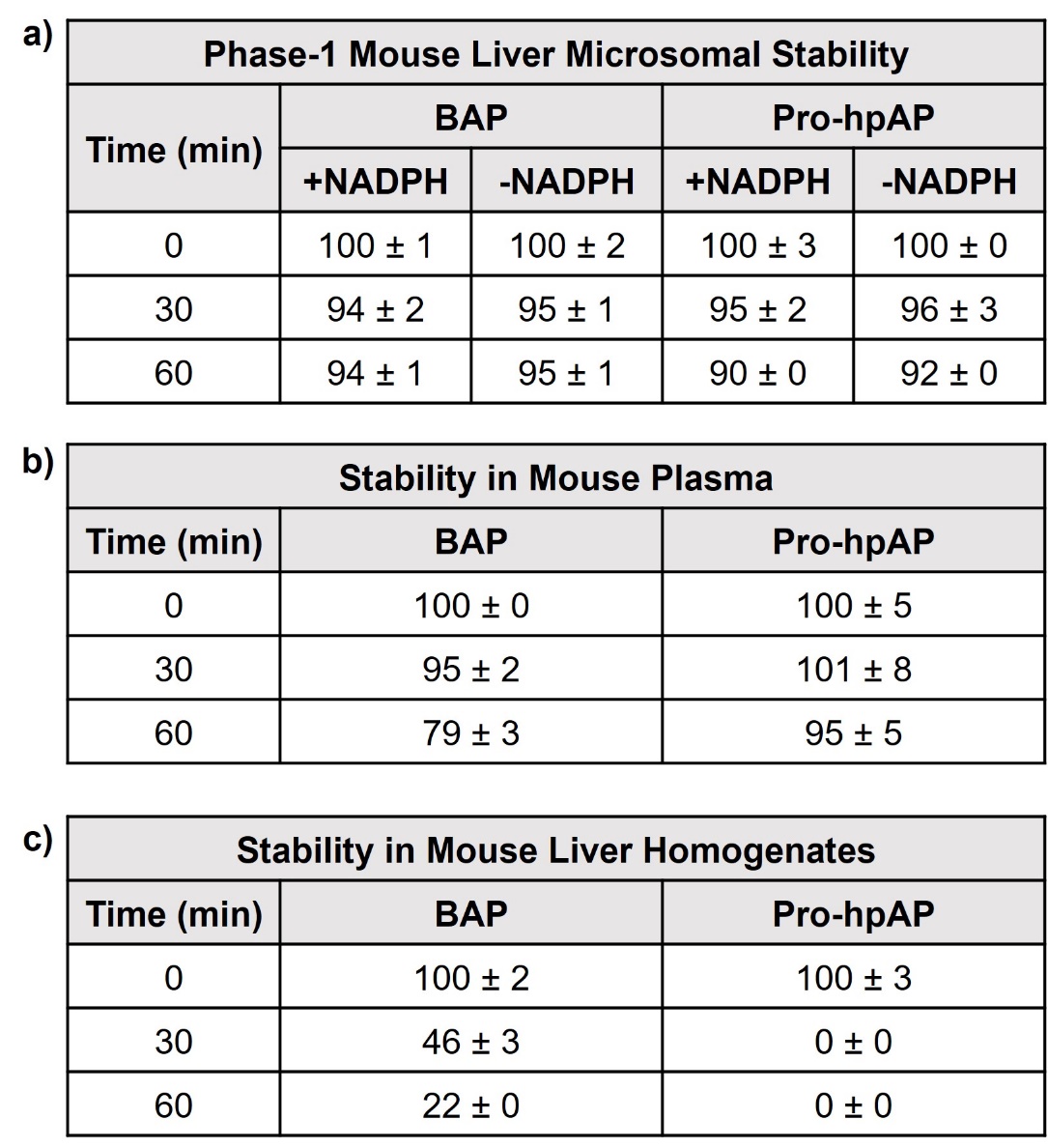


**Figure S1.** Percent BAP or pro-hpAP remaining after incubation in mouse liver microsomes (+/-NADPH) (**a**), mouse plasma (**b**), or mouse liver homogenates (**c**). Data shown as bar graphs in Figure 4.


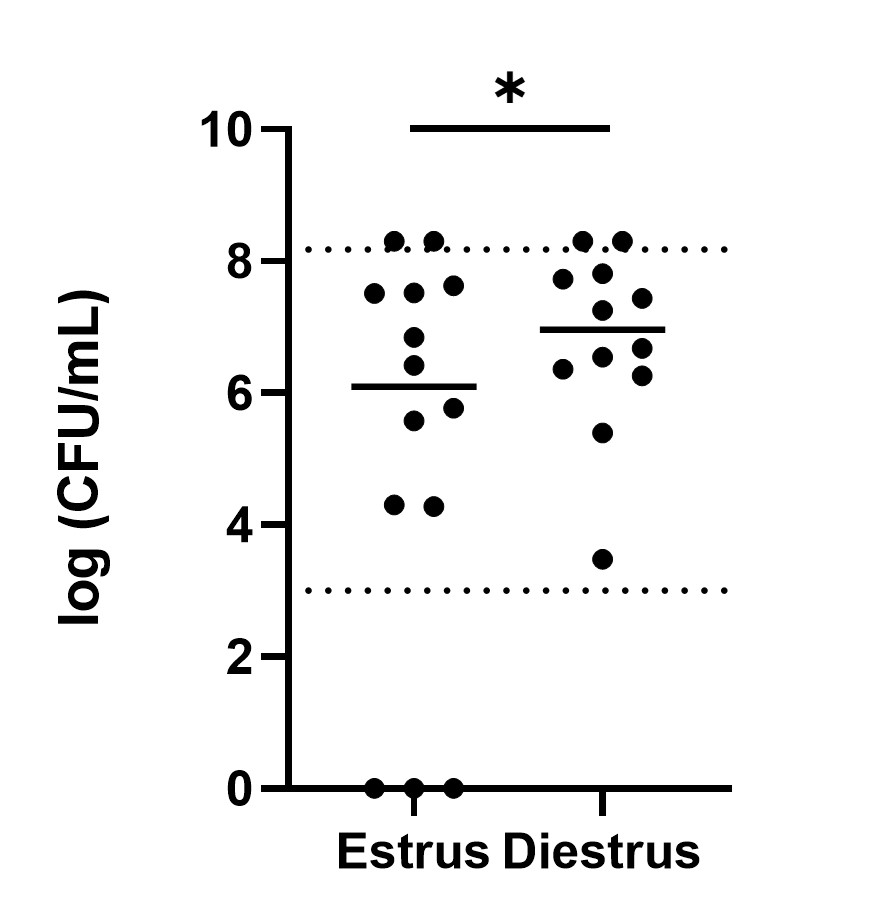


**Figure S2**. Bacterial burden after transurethral inoculation correlated to the natural estrous cycle of untreated mice at the time of catheterization. Mice at different points in the estrous cycle have different variances as indicated by F-test. Each point depicts an individual mouse, with the line representing median log (CFU/mL); F test for variance, p ≤ 0.05 (*). Dotted lines represent limits of detection.


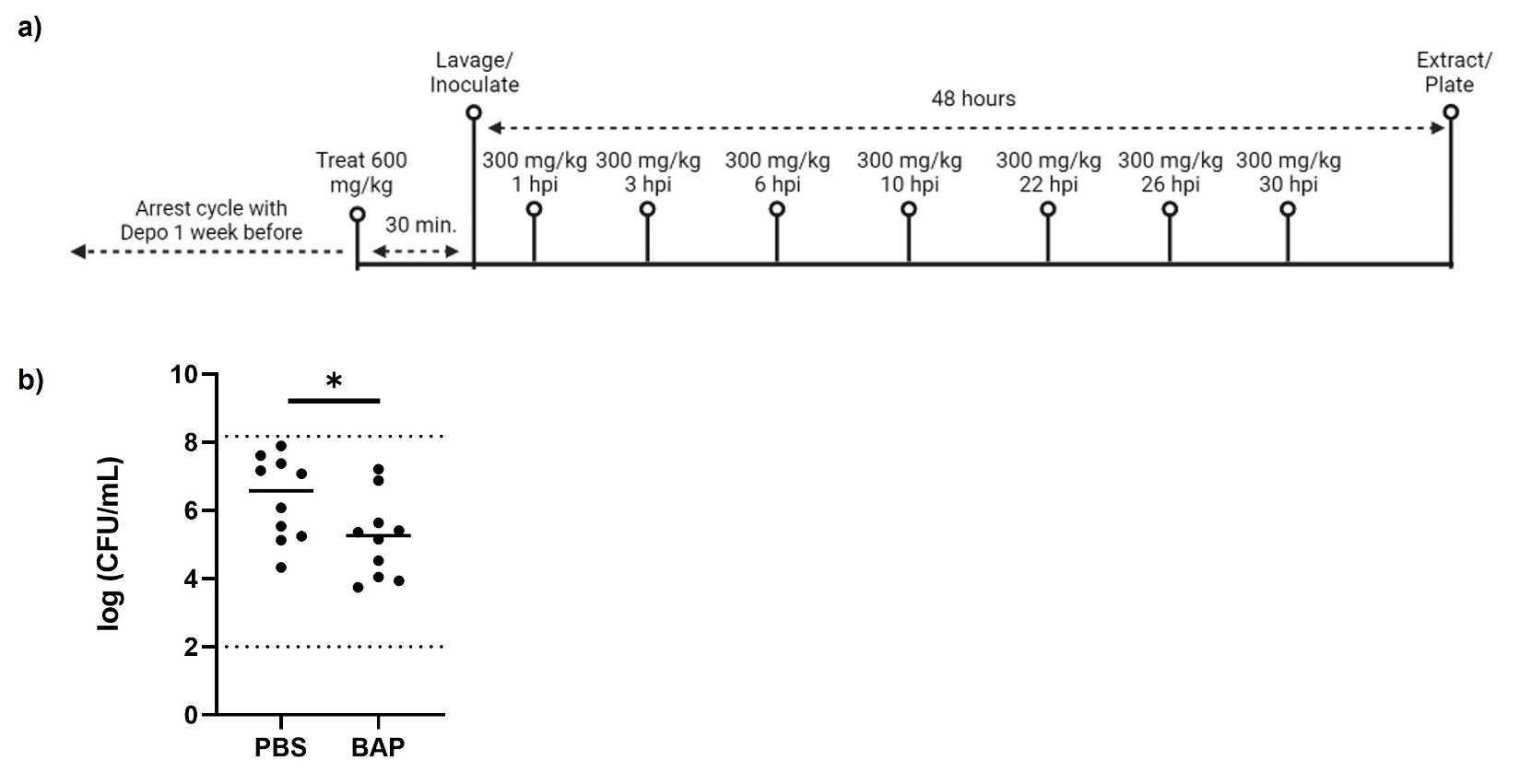


**Figure S3**. Additional doses of BAP after 3 hours reduce bacterial burden 1.32 log relative to no-drug control and (**a**) provide no increased benefit in protecting mice from UTI (**b**). Each point depicts an individual mouse (**b**), with the line representing median log (CFU/mL); p ≤ 0.05 (*). Dotted lines represent limits of detection.
